# Urinary Extracellular Domain of Neurotrophin Receptor p75 as a Biomarker for Amyotrophic Lateral Sclerosis in a Chinese cohort

**DOI:** 10.1038/s41598-017-05430-w

**Published:** 2017-07-11

**Authors:** Rui Jia, Stephanie Shepheard, Jiaoting Jin, Fangfang Hu, Xing Zhao, Li Xue, Li Xiang, Huaguang Qi, Qiumin Qu, Feng Guo, Mary-Louise Rogers, Jingxia Dang

**Affiliations:** 1 Department of Neurology, The First Affiliated Hospital of Xi’an Jiaotong University, 277 Western Yanta Rd, Xi’an, 710061 China; 2 Department of Human Physiology and Centre for Neuroscience, Flinders University, Adelaide, South Australia Australia; 3 Department of Laboratory, The Second Affiliated Hospital of Xi’an Jiaotong University, 157 Xiwu Rd, Xi’an, China; 4 EMG Lab, The Second Affiliated Hospital of Xi’an Jiaotong University, Xi’an, P.R. China; 5 EMG Lab, Xi’an Red Cross Hospital, 555 East of Youyi Rd, Xi’an, China

## Abstract

To comprehensively assess whether p75^ECD^ in urine could be a candidate biomarker for ALS evaluation. Urine samples were collected from 101 ALS patients, 108 patients with other neurological disease (OND) and 97 healthy controls. 61 ALS patients were followed up with clinical data including ALSFRS-r every 6 to 12 months, 23 ALS patients died and 17 ALS patients lost touch during follow up period. Enzyme-linked immunoassay was employed to determine urine p75^ECD^ concentration. The ALSFRS-r was employed to assess the severity of ALS. The concentration of p75^ECD^ in ALS was significantly higher than that of OND and CTRL (p < 0.001). Additionally, urine p75^ECD^ concentrations in ALS-definite grade patients were significantly higher than that in ALS-probable grade and ALS-possible grade patients (p < 0.001). Higher urine p75^ECD^ concentrations were correlated with increased clinical stage (p = 0.0309); urine p75^ECD^ concentrations and ALSFRS-r were negatively correlated (p = 0.022); and urine p75^ECD^ concentration in the fast-progressing ALS group was significantly higher than that in slow-progression (p = 0.0026). Our finding indicates that urine p75^ECD^ concentration provides additional evidence for patients with clinically suspected ALS, and can be employed to evaluate ALS-severity.

## Introduction

Amyotrophic lateral sclerosis (ALS) is a rapidly progressive, fatal neurodegenerative disorder affecting upper (UMN) and lower (LMN) motor neurons in the brain and spinal cord^1^, which usually causes death due to respiratory muscle paralysis within 3 years of onset^2^. Whilst the prevalence and incidence of ALS is anecdotally similar across countries, it is not fully described in China^3^. However, a recent study concluded China is predicted to have similar incidence and prevalence to the western world, and the number of individuals with ALS will grow significantly in China between 2015 and 2040^4^. ALS diagnosis is usually based on clinical assessment and electrophysiological examination^5^, and there may be a pronounced delay between the onset of symptoms and diagnosis^6^. ALS progression is assessed in the clinic by the revised ALS functional rating scale (ALSFRS-r)^7^. This is based on a patient questionnaire. However, additional objective biomarkers that can supplement neurological data may improve discrimination of ALS patients from other probable diseases and help to evaluate progression.

Since there are no effective treatments for ALS, biomarkers that can change with disease (progression) and improve stratification of trials by reflecting disease severity (i.e. are prognostic), are useful in clinical trials of ALS treatments^8^. Neurofilaments (heavy and light chains) in blood and CSF have shown potential as prognostic biomarkers^9–11^. Notably, we have recently shown that the extracellular domain of the common neurotrophin receptor p75 (p75^ECD^) has potential as a progression and prognostic marker for ALS^12^. Urinary p75^ECD^ is the first fluid based ALS biomarker of disease progression.

We firstly showed urinary p75^ECD^ was higher in ALS than healthy controls and other disease (Parkinson’s and Multiple Sclerosis)^13^. In a follow up study that encompassed samples from Australia and the USA, urinary p75^ECD^ was again shown to be higher in ALS (n = 54 patients) than controls (n = 45). Notably, urinary p75^ECD^ increased over time with disease progression in 31 ALS patients, sampled a median of 2 time points over 2–3 years^12^. In addition, we showed in cross-sectional data it had prognostic value, over and above for example site of onset. We now wish to validate our finding that p75^ECD^ is a potential biomarker of ALS and disease severity-evaluation, in, a larger Chinese cohort.

## Results

### Participant Characteristics

Urine samples were collected from three groups: 101 people with ALS, 108 people with OND and 97 CTRL. There were 61 ALS patients assessed by ALSFRS-r every 6 to 12 months, 23 ALS patients died during the follow up and 17 ALS patients lost follow up. The demographic characteristics of the three groups are shown in Table 1.

**Table 1:** Demographic Characteristic of ALS, OND and CTRL.

Variable	ALS	OND	CTRL
Age at diagnosis (Mean ± SD)	54.78 ± 10.61^a^	55.95 ± 13.19^a^	56.81 ± 11.98^a^
Gender (M/F)	57/44^b^	60/48^b^	56/41^b^
Bulbar onset (%)	16 patients (15.84)	—	—
Months from onset to diagnosis (Mean ± SD)	15.3 ± 12.85	—	—
ALSFRS-r at baseline	38.50 ± 6.41	—	—
ALSFRS-r at last follow up	29.64 ± 9.00		
Death by end of study	23 patients	—	—
Months from diagnosis to death (Mean ± SD)	10.1 ± 4.38	—	—

^a^ and ^b^: there was no difference in age or gender among three groups (p > 0.05).

### Urine p75^ECD^ concentrations in ALS patients

**Figure 1:**
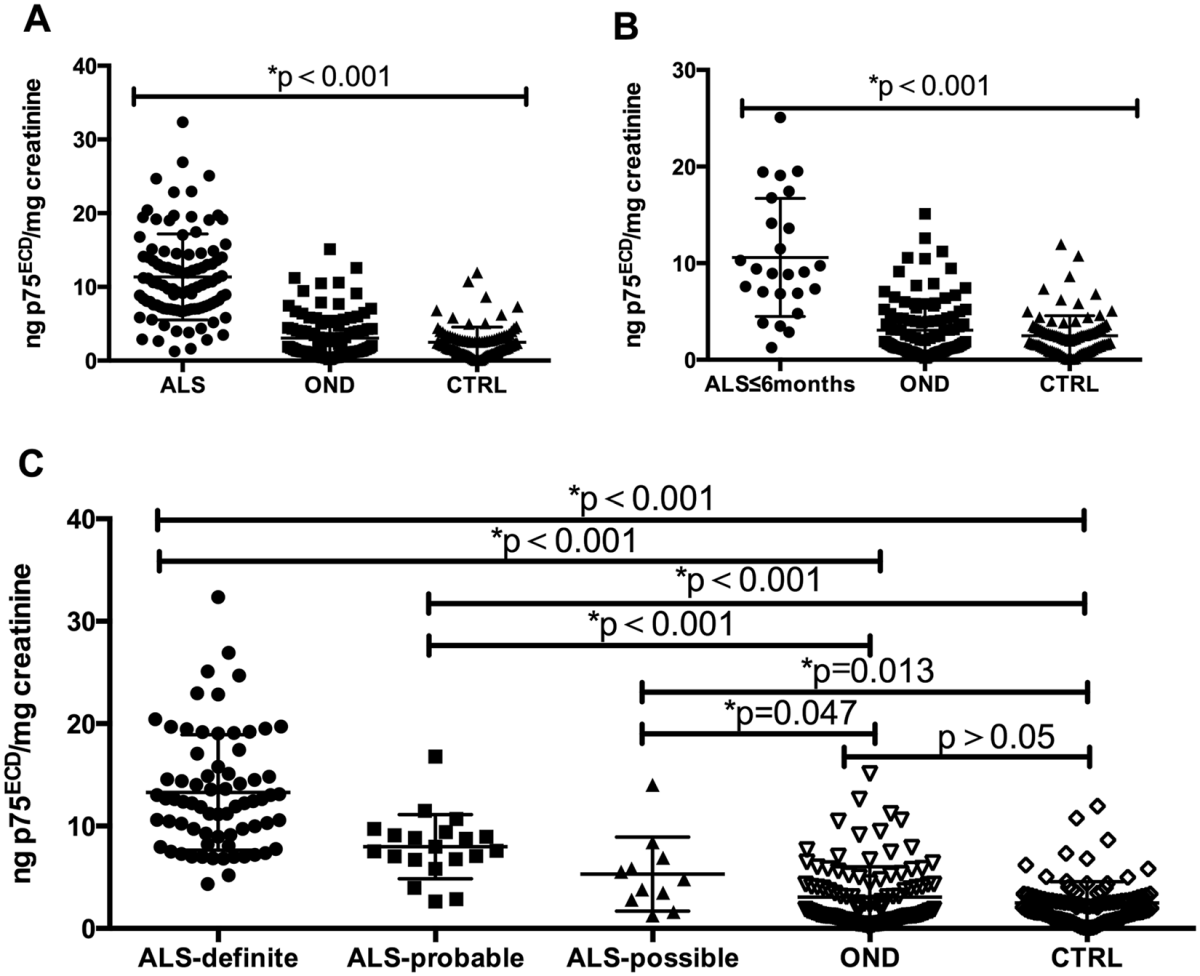
Comparison of p75^ECD^ in urine between different groups and ALS Diagnostic Grades: (**A**) Urine p75^ECD^ in ALS was higher than OND and CTRL by one way ANOVA (p < 0.001). (**B**) Urine p75^ECD^ in ALS (onset less than 6 months) was higher than OND and CTRL by one way ANOVA (p < 0.001). (**C**) Urine p75^ECD^ was different among three ALS diagnosis grades (p < 0.001), and the rank test among them suggests that urine p75^ECD^ increased with ALS diagnostic degree (p < 0.001).

The urine p75^ECD^ concentrations in (n = 101) patients with ALS (11.36 ± 5.83 ng p75^ECD^/mg creatinine) was significantly higher than that of OND (stroke, Parkinson’s and Multiple Sclerosis) (3.06 ± 2.95 ng p75^ECD^/mg creatinine) and CTRL (2.49 ± 2.07 ng p75^ECD^/mg creatinine, p < 0.001), as shown in Fig. 1A. Additionally, the urine p75^ECD^ concentration in patients with an onset-time less than 6 months (10.59 ± 6.11 ng p75^ECD^/mg creatinine, n = 25) was significantly higher than that of OND and CTRL (p < 0.001, Fig. 1B).

We further divided the ALS group into three diagnostic grades: clinically definite (n = 70), clinically probable (n = 20) and clinically possible grade (n = 11), based on the revised El Escorial criteria^14^. The p75^ECD^ concentration in clinically definite grade patients (13.27 ± 5.64 ng p75^ECD^/mg creatinine) was significantly higher than that in clinically probable grade (7.99 ± 3.13 ng p75^ECD^/mg creatinine) and clinically possible grade (5.32 ± 3.61 ng p75^ECD^/mg creatinine, p < 0.001) by one way ANOVA. The LSD test showed that the urine p75^ECD^ concentrations of the three ALS diagnostic grade were significantly higher than that of OND or CTRL, respectively (ALS- Definite *VS* OND, p < 0.001; ALS- Definite VS CTRL, p < 0.001; ALS- Probable VS OND, p < 0.001; ALS- Probable VS CTRL, p < 0.001; ALS- Possible VS OND, P = 0.047; ALS- Possible VS CTRL, p = 0.013; Fig. 1C). It should also be noted that there was no obvious difference in p75^ECD^ levels between OND and CTRL groups (p > 0.05; Fig. 1C).

### Urine p75^ECD^ concentrations and diagnostic Level

**Figure 2:**
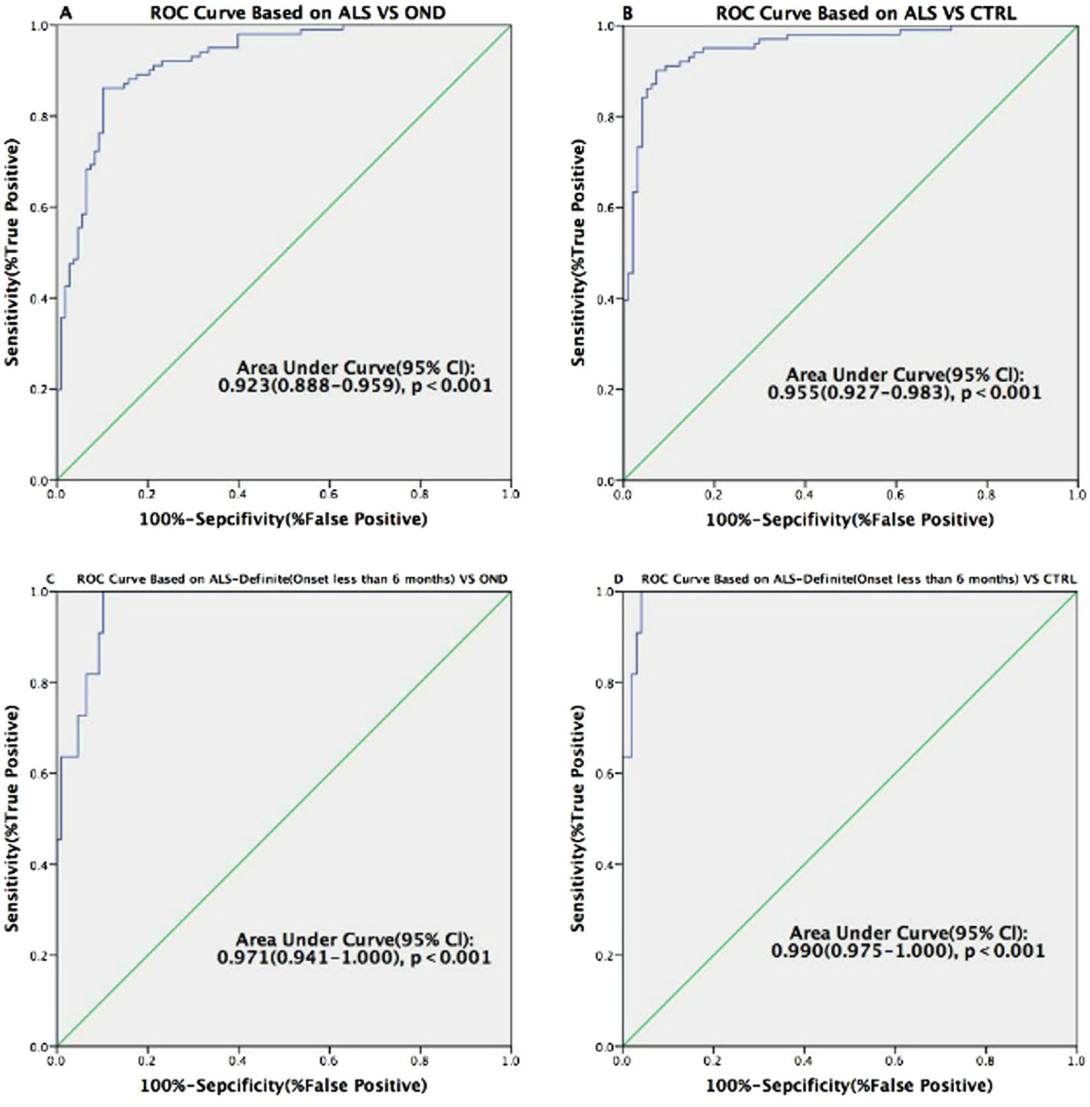
Receiver Operating Characteristic curves for distinguishing ALS/ALS-definite patients (onset less than 6 months) from OND and CTRL. (**A**) ALS was distinguished from OND with 6.6945 (ng p75^ECD^/mg creatinine) as the cuff-off value; (**B**) ALS was distinguished from CTRL with 5.1221 (ng p75^ECD^/mg creatinine) as the cut off value. (**C**) ALS-definite patients less than 6 months were distinguished from OND with 7.2015 (ng p75^ECD^/mg creatinine) as the cut-off value; (**D**) ALS-definite patients less than 6 months were distinguished from CTRL with 6.827 (ng p75^ECD^/mg creatinine) as the cut off value.

In order to evaluate urine p75^ECD^ concentrations among the different diagnostic levels, receiver operating characteristic (ROC) curves were generated from ALS VS CTRL and OND data, respectively. Urine p75^ECD^ concentrations can distinguish ALS patients from OND, with sensitivity of 86.1%, specificity of 89.8%, and area under the curve (AUC) of 0.923 (95% confidence limits of area 0.888–0.959; Fig. 2A), so does CTRL, with sensitivity of 90.1%, specificity of 92.8%, and AUC of 0.955 (95% confidence limits of area 0.927–0.983; Fig. 2B).

The ability to use p75^ECD^ to identify ALS-definite patients (onset less than 6 months) from OND, was determined using ROC analysis (Fig. 2C), showing 7.2015 (ng p75^ECD^/mg creatinine) as the cuf-off value; sensitivity of 90.9%; specificity of 90.7%; AUC of 0.971 (95% confidence limits of area 0.941–1.000, p < 0.001). Meanwhile, using urine p75^ECD^ concentrations to distinguish ALS-definite patients less than 6 months from CTRL, showed 6.827 (ng p75^ECD^/mg creatinine) as the cut-off value; sensitivity: 100%, specificity: 95.9%, and AUC: 0.990 (95% confidence limits of area 0.975–1.000, p < 0.001; Fig. 2D).

**Figure 3:**
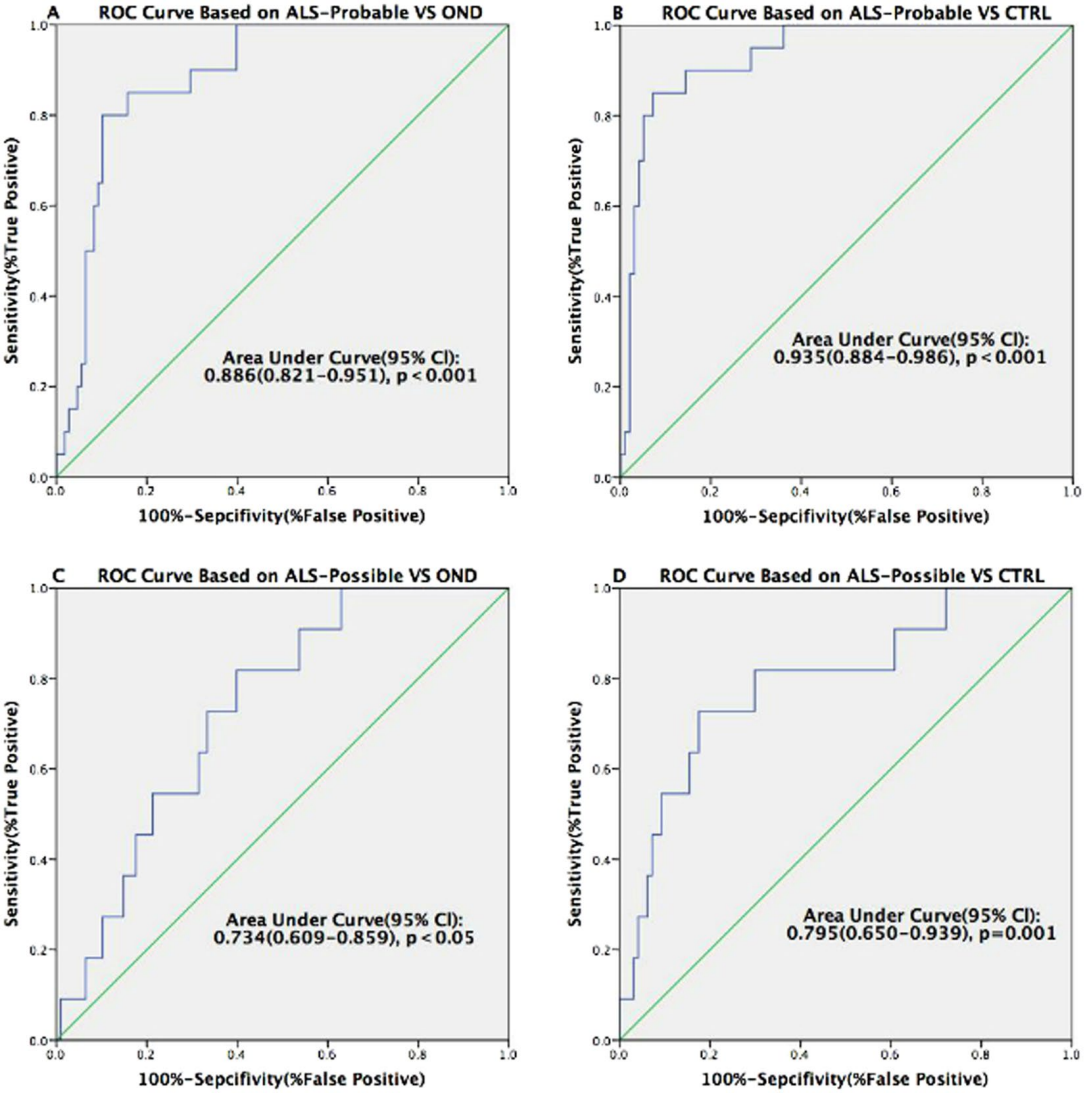
Receiver Operating Characteristic curves for distinguishing ALS-Probable/Possible patients from OND and CTRL. (**A**) The p75^ECD^ cut-off value was 6.6945 (ng p75^ECD^/mg creatinine) between ALS-probable patients and OND; (**B**) The p75^ECD^ cut off value was 5.4301 (ng p75^ECD^/mg creatinine) between ALS-probable patients and CTRL. (**C**) The p75^ECD^ cut-off value was 2.7201 (ng p75^ECD^/mg creatinine) between ALS-possible patients and OND; (**D**) The p75^ECD^ cut-off value was 3.4685 (ng p75^ECD^/mg creatinine) between ALS-possible patients and CTRL.

ALS-probable patients were also distinguishable from OND/CTRL with a sensitivity of 80.0%/85.0%, specificity of 89.8%/92.8%, and AUC 0.886/0.935 respectively (ROC analysis, Fig. 3A,B). The ROC also showed that ALS-possible patients were distinguished from OND/CTRL with sensitivity of 81.8%/72.7%, specificity of 60.2%/82.5%, and AUC 0.734/0.795 respectively (Fig. 3C,D).

### Urine p75^ECD^ concentrations with clinical stage

**Figure 4:**
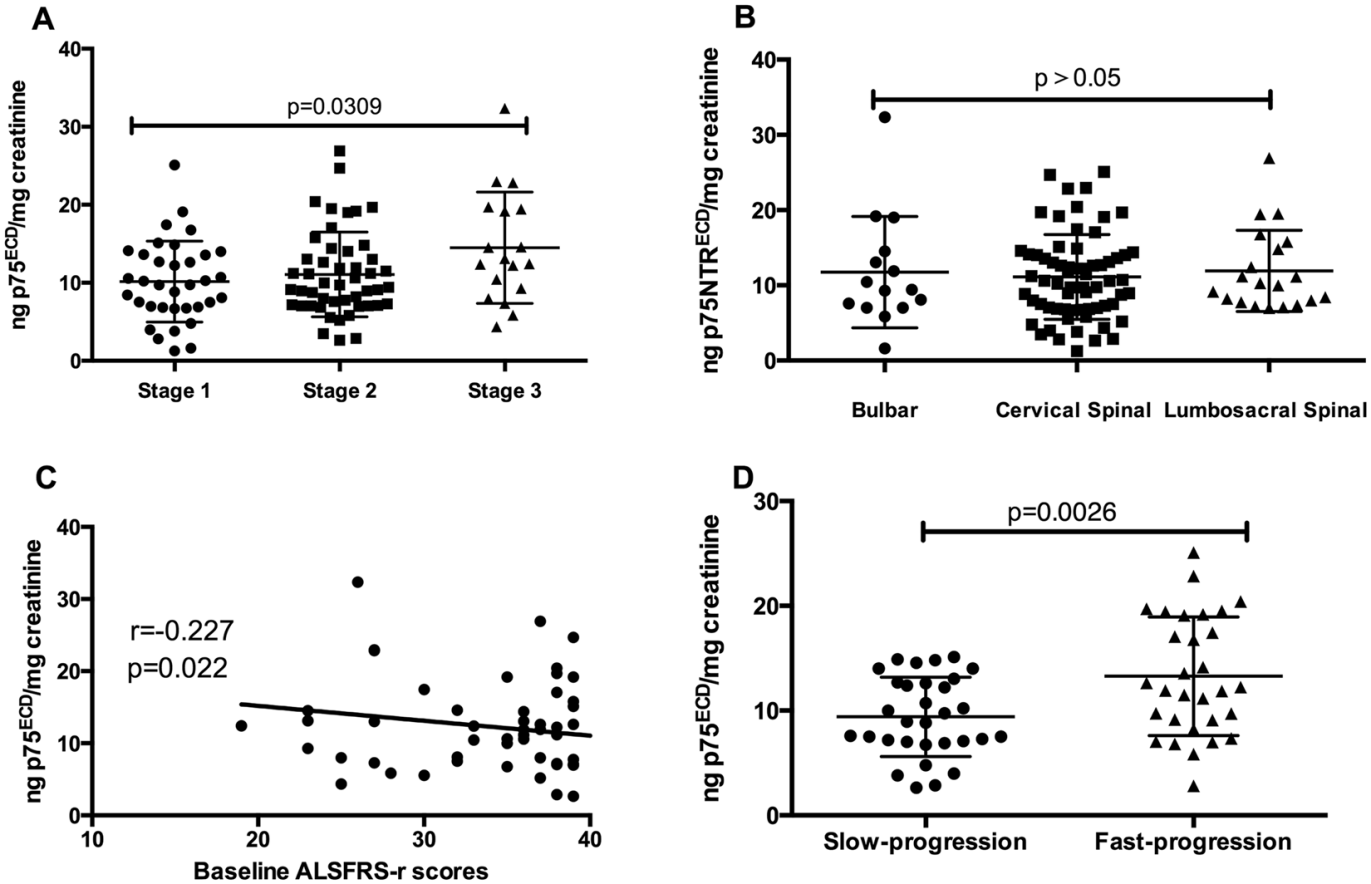
Urine p75^ECD^ levels and ALS-severity and progression. (**A**) There was significant difference among three clinical stages; (**B**) There were no differences in urine p75^ECD^ concentrations among ALS patients with different onset regions; (**C**) There was negative correlation between baseline p75^ECD^ and ALSFRS-r scores in ALS patients; (**D**) Significantly higher urine p75^ECD^ concentrations were detected in the fast-progression group than in the slow-progression group.

Using the King’s College clinical stage for ALS^15^, we have divided patients into stage 1, stage 2 and stage 3. Stage 1 is the ALS patients involved one region, stage 2 is involved two regions, stage 3 is involved three regions, stage 4 is when gastrostomy or non-invasive ventilation is required, stage 5 is death. We then analyzed urine p75^ECD^ in different stages, and it showed a significant increase level from 1–3 (p = 0.0309; Fig. 4A). Urine p75^ECD^ concentration was 10.15 ± 5.17 ng p75^ECD^/mg creatinine in stage 1(n = 35), stage 2(n = 48) was 11.07 ± 5.43 ng p75^ECD^/mg creatinine and stage 3(n = 18) was 14.49 ± 7.13 ng p75^ECD^/mg creatinine. There were no ALS patients reached stage 4 or 5 at the baseline. Urine p75^ECD^ concentrations did not differ between patients with different onset sites. Patients with lumbosacral spinal cord vs bulbar, or lumbosacral spinal cord vs cervical spinal cord, or cervical spinal cord vs bulbar onset disease were not significantly different (Bulbar: 11.75 ± 7.41 ng p75^ECD^/mg creatinine, n = 15; cervical spinal cord: 11.10 ± 5.63 ng p75^ECD^/mg creatinine, n = 66; lumbosacral spinal cord: 11.92 ± 5.39 ng p75^ECD^/mg creatinine, n = 20; p > 0.05; Fig. 4B).

### Association of urine p75^ECD^ concentration with progression rate

There was a negative correlation between urine p75^ECD^ concentration and ALSFRS-r scores in ALS patients at first collection (r = −0.227, p = 0.022; Fig. 4C, n = 101).

All ALS patients were given follow up visits during the research, and 61 of 101 ALS patients were assessed by ALSFRS-r every 6 to 12 months. The progression rate (Δr) was calculated as the monthly ALSFRS-r score slope in the time interval between the time of sampling and the last follow-up visit^16^. According to the median progression rate of 0.67^10^, all ALS patients were divided into two groups: fast-progression (Δr > 0.67, 13.28 ± 1.035 ng p75^ECD^/mg creatinine, n = 30), and slow-progression (Δr ≤ 0.67, 9.410 ± 0.6804 ng p75^ECD^/mg creatinine, n = 31). It was found that, urine p75^ECD^ concentrations in the fast-progression group were significantly higher than that in slow-progression (p = 0.0026; Fig. 4D).

## Discussion

This study supports urinary p75^ECD^ as a biomarker for people with ALS. In our previous studies, urine p75^ECD^ concentrations were significantly higher in ALS patients than normal controls^12^ and patients with PD or MS^13^. Our current study confirms this finding, but in a Chinese cohort. Interestingly, Chinese ALS patients have a higher baseline level of p75^ECD^ when compared to Australian/USA patients (11.36 ± 5.83 versus 5.6 ± 2.2)^12^. Healthy controls in the Chinese cohort (2.49 ± 2.07) had similar levels to Australian/USA patients (3.6 ± 1.4). There is little literature on differences between Chinese and other racial groups as to disease severity at onset and should be investigated further.

Our data indicates that urinary p75^ECD^ may add value to diagnostic evidence for the patients with clinically suspected ALS. In addition, urinary p75^ECD^ concentration increases with diagnostic grades of ALS indicating urinary p75^ECD^ may be increasing with amount of lesion sites and the severity. Further, there was a negative correlation between baseline ALSFRS-r and urinary p75^ECD^. This goes somewhat to validating our previous work that showed urinary p75^ECD^ is a marker of severity^12^.

Our results also indicate a significant elevation in p75^ECD^ concentrations in ALS patients compared to those with stroke, Parkinson’s and multiple sclerosis. Previously we showed Parkinson’s and multiple sclerosis (as other neurological diseases) was elevated, but not significantly higher than controls or ALS patients^13^. In this current study, ROC analysis showed some discrimination of ALS from OND. However, it should be noted that we will in future work address specificity by examining other related conditions such as FTD, and mimics’ such as neuropathy, Kennedy’s syndrome and primary lateral sclerosis. We do not expect elevated urinary p75^ECD^ to be highly specific for ALS. p75^ECD^ is well known to be elevated in nerve injury^17^. We and others have previously shown p75^ECD^ is up-regulated on motor neurons in MND mice models^18, 19^, and in ALS^13, 20^. Since we wish to develop this biomarker as a progression marker that can be of use in clinical trials, specificity is not an absolute requirement. For example, elevation in CSF and blood neurofilament heavy (NFH) and light chains (NfL) is not specific to ALS, yet they are well developed as prognostic biomarkers for ALS. Further, NfL levels in blood and CSF, are largely stable over time for periods of up to 15-months of follow-up^21^. A recent larger study of ALS patients confirmed NfL in CSF is a useful prognostic biomarker, and for example may help define subgroups, as it is higher in frontal-temporal dementia^22^. Hence, even if urinary p75^ECD^ is raised in other diseases, we suggest it is a valid biomarker for ALS, notably for clinical trials of treatments.

Due to its convenience to obtain, urinary biomarkers have advantages over for example CSF. Most ALS patients are willing to provide urine, but not all CSF, it is thus an easily accessible biomarker source^23^. ELISA assays for fluid based biomarkers can also be cost effective when compared to imaging biomarker; an important point to consider in some areas in the world.

We found urinary p75^ECD^ levels in a fast-progressing ALS group were significantly higher than that in a slow-progression group and there was a negative correlation between p75^ECD^ and ALSFRS-r scores in ALS patients. This suggests that the p75^ECD^ levels in ALS patients could be employed to reflect the disease process and point towards fast progression rates. This again suggests that urinary p75^ECD^ is a marker of disease progression in ALS and agrees with our previous study^12^. Hence this validates this marker to be investigated as a pharmacodynamic marker to test the effectiveness of therapies in clinical trials.

Our study is not without its drawbacks. Most notably, the study population in China may not be as defined as in other reported studies. Moreover, the limited number of samples and assessments available from each patient precluded measuring p75^ECD^ in consecutive samples and progression rate (as we have done previously). These shortcomings, are being addressed in an on going study.

In conclusion, we provide further evidence that urinary p75^ECD^ concentration could be used as biomarker for ALS. We found that urinary p75^ECD^ concentration reflects ALS severity, and supplies additional evidence for patients with clinically suspected ALS. The easily accessible, non-invasive characteristics make urine highly practical as a biomarker tool. Further studies on the comparison of urine p75^ECD^ for ALS and other ALS-similar diseases should be employed to verify the validity for ALS diagnosis, as well as the change of urine p75^ECD^ in the longitudinal follow-up.

## Materials and Methods

### Participants and samples collection

Participants were divided into three groups: the ALS group (ALS), other neurological disorders group (OND) and normal control group (CTRL), which were age and gender matched. Urine samples from ALS patients were collected at first diagnosis in the Neurology Department of the First Affiliated Hospital of Xi’an Jiaotong University, the Second Affiliated Hospital of Xi’an Jiaotong University, and Xi’an Red Cross Hospital from July 2014 to October 2015; all ALS patients were followed up with clinical data including ALSFRS-r every 6 to 12 months. ALS patients were strictly diagnosed by at least two experienced neurologists according to the revised El Escorial criteria, and further divided into three diagnostic grades: clinically definite, defined on clinical evidence alone by the presence of UMN and LMN signs in at least three regions; clinically probable, the presence of UMN and LMN signs in at least two regions with some UMN signs necessarily rostral to (above) the LMN signs; clinically possible, clinical signs of UMN and LMN dysfunction are found together in only one region or UMN signs are found alone in two or more regions, or LMN signs are found rostral to UMN signs^14^. The clinical stages of ALS patients were based on the King’s College clinical stage for ALS^15^. p75^ECD^ is expressed in both nervous tissues and cancerous tissues^24^, so in the ALS group, there were no evidence of cancer, their medical history and the results of electromyography were recorded in detail. The progression rate (Δr) was calculated as the monthly ALSFRS-r score slope in the time interval between the time of sampling and the last follow-up visit^16^. The OND group consisted of patients with common neurological diseases in which the concentration of p75^ECD^ was unknown - acute ischemic/hemorrhage stroke (confirmed by CT/MRI scans within one week from the symptom onset), Parkinson’s Disease (PD; based on the UK Parkinson’s Disease Society Brain Bank^25^ criteria), or Multiple Sclerosis (MS; according to the revised McDonald diagnostic criteria^26^) The participants in the CTRL group were from healthy volunteers without nervous system diseases. Urine samples from all participants were collected according to the Urine and Kidney Proteome Project Standards^27^. This study was evaluated and approved by the Ethics Committee of the First Affiliated Hospital of Xi’an Jiaotong University, with signed informed consent provided by all participants.

### Measurement of p75^ECD^ in Urine by ELISA

Each urine sample was tested in triplicate to quantify p75^ECD^ by sandwich ELISA. Anti-p75^ECD^ MLR1^27^ antibody for coating ELISA plates was provided by Flinders University. In our experiments, Anti-p75^ECD^ MLR1 (4 μg/ml, 100 μl/well) in coating buffer (25 mM Na_2_CO_3_, 25 mM NaHCO_3_, 0.01% Thimerosal, pH 9.6) was used to coat for 18 hours at 4 °C in 96-wells plates (Costar Corning). Then, sample buffer (5% 20xPBS, 2% BSA, 0.05% Tween-20, 0.01% Thimerosal, pH 7.4) was used to block wells for 1 hour at 37 °C. Recombinant human p75^ECD^ (R&D systems, 367-NR-050) was used for the standard curve, and urine samples diluted in sample buffer were incubated for 20 hours at room temperature with gentle agitation. After washing, mouse anti-p75^ECD^ (R&D systems, AF1157) (1 μg/ml, 100 μl/well) diluted in sample buffer was used as the detection antibody for one hour at room temperature with gentle agitation. Secondary antibody (bovine anti Goat IgG-HRP; Jackson ImmunoResearch, 805-035-180) (0.8 μg/ml, 100 μl/well) was used to mark the detecting antibody for one hour at room temperature with gentle agitation. TMB (Life technologies, 00-2023) was used for the peroxidase reaction for 15 minutes, then 2 M sulphuric acid was added to stop the colour reaction. Absorbance values were read at 450 nm using a 96-well microplate reader (Thermo Systems, Boston, MA, USA). Wash buffer (5% 20xPBS, 0.05% Tween-20, pH 7.4) was used to wash 96-wells for four washes between steps. Urinary creatinine was measured by Department of Laboratory in the Second Affiliated Hospital of Xi’an Jiaotong University using enzymatic analysis.

### Statistical analysis

Urine p75^ECD^ levels among different groups was analyzed by one-way ANOVA after Bonferroni’s multiple comparison in Prism 5. The difference in p75^ECD^ concentration in urine among three ALS diagnosis grades and CTRL was analyzed by rank test. The sensitivity and specificity of the diagnosis for ALS were tested using Receiver Operating Characteristic (ROC) curve analysis using SPSS, and the Youden Index was used to calculate cut-off levels for ALS.
